# The Genetic Basis of Natural Variation in Kernel Size and Related Traits Using a Four-Way Cross Population in Maize

**DOI:** 10.1371/journal.pone.0153428

**Published:** 2016-04-12

**Authors:** Jiafa Chen, Luyan Zhang, Songtao Liu, Zhimin Li, Rongrong Huang, Yongming Li, Hongliang Cheng, Xiantang Li, Bo Zhou, Suowei Wu, Wei Chen, Jianyu Wu, Junqiang Ding

**Affiliations:** 1 College of Agronomy, Synergetic Innovation Center of Henan Grain Crops and National Key Laboratory of Wheat and Maize Crop Science, Henan Agricultural University, Zhengzhou, 450002, China; 2 The National Key Facility for Crop Gene Resources and Genetic Improvement, Institute of Crop Science and CIMMYT China Office, Chinese Academy of Agricultural Sciences, Beijing 100081, China; 3 Henan Vocational College of Agriculture, Zhengzhou, 450002, China; National Key Laboratory of Crop Genetic Improvement, CHINA

## Abstract

Kernel size is an important component of grain yield in maize breeding programs. To extend the understanding on the genetic basis of kernel size traits (i.e., kernel length, kernel width and kernel thickness), we developed a set of four-way cross mapping population derived from four maize inbred lines with varied kernel sizes. In the present study, we investigated the genetic basis of natural variation in seed size and other components of maize yield (e.g., hundred kernel weight, number of rows per ear, number of kernels per row). In total, ten QTL affecting kernel size were identified, three of which (two for kernel length and one for kernel width) had stable expression in other components of maize yield. The possible genetic mechanism behind the trade-off of kernel size and yield components was discussed.

## Introduction

Maize (*Zea mays* L.) is one of the most important cereal crops in the world, and increasing the maize production by selection for the components of grain yield is the main objective in maize breeding programs [1]. Maize kernel size, measured by kernel length, width and thickness, is an important component of grain yield. Moreover, the characteristic of kernel size is also an important factor of appearance quality, which may influence the corn market grades and consumer preference [2]. Therefore, investigating the genetic basis of kernel size, and discovering any possible genetic constraints to optimize it, will facilitate the improvement of grain yield in maize breeding programs.

Quantitative trait locus (QTL) mapping based on molecular markers have been widely used in the genetic study for different traits in different crops [3–6]. There were several QTL/genes have been identified in grain crops for kernel size [2,7–9]. Especially, some genes for kernel traits have been successfully isolated by map-based cloning strategy in rice, *e*.*g*., *GS3* [3,10], *qGL3* [11], *GW2* [4], *qSW5* [12], *GS5* [13], *GW8* [14], *GL7* [15] and *GW7* [16], and these genes can be useful targets of molecular-assisted selection for larger seed size in modern rice breeding programs. In maize, mutants analysis have identified several genes in key pathways involved in seed development, such as *Mn1* [17], *o2* [18], *sh2* [19], *gln1-4* [20], *o1* [21], and others [22]. Compared with the effects of mutants in kernel size, which are often dependent on the genetic background [23], QTL mapping approach is ideal to identify favorable QTL/genes that can contribute to natural variation of kernel size. In recent years, mapping QTL for kernel size is attractive in maize, and most studies for kernel size and related traits have been conducted in bi-parental populations [7–9,24–28].

The available QTL generally detected in bi-parental mapping populations have greatly contributed to the understanding of the genetic basis of kernel size. However, QTL mapping in such populations is subject to low allele numbers and limited recombination [29]. In recent years, the generation of multi-parent advanced generation integrated cross (MAGIC) populations has provided an additional option for QTL mapping. Compared with the bi-parental linkage populations, the development of MAGIC populations usually involved inter-crossing of multiple parental lines, which may introduce more than two independent alleles at a locus and subsequently increased probability of QTL being polymorphic across the multiple parents [30]. In addition, the precision and resolution of QTL detection can be increased by the amplified number of recombination events [31]. In view of the merits of MAGIC for QTL mapping, increasing number of MAGIC populations have been created in model animals and plants recently. For example, two MAGIC populations have been developed in mice and used for identifying candidate genes for serum cholesterol and coat color traits [32,33]. In plants, MAGIC populations were first developed in Arabidopsis and subsequently expanded to crops [34]. In recent years, encouraging results have been reported for flowering time, leaf morphology and seed traits of Arabidopsis thaliana [35–37], fruit weight of tomato [38], plant height and shoot traits of wheat [39,40], biotic stress and abiotic stress of rice [41] and flowering time of barley [42]. Very recently, MAGIC populations were also developed in maize and then used for QTL mapping in traits such as flowering time, plant height, ear height and grain yield [43].

Crop seed is a life-history trait, and the availability of resource pool in seed developmental processes drives seed production [2,44,45]. Due to the competing apportionment of resources between fitness components (i.e., seed size and seed number), a trade-off between seed number and size must occur [46]. A better understanding of natural variation in seed size requires simultaneous consideration of trade-off of kernel number related traits to seed development [37].

Given the potential benefits of multi-parental (four-way cross) mapping population, we developed a set of multi-parental (four-way cross) mapping population in maize [6]. In the present study, we investigated the natural variation in seed size and other seed related traits. The objectives of this study were to detect the genetic architecture underlying seed size in maize, and specifically we were interested in the genetic mechanism behind trade-off of seed traits to better understand the genetic basis of kernel size.

## Materials and Methods

The experiment was conducted in Zhengzhou Experiment Station (34°51'N 113°35'E) and Jiyuan Experiment Station (35°4'N 112°36'E) of Henan Agricultural University (HAU). At the two experimental locations, HAU has set up experimental field bases for non-profit agricultural research with a wide array of partners in China. In the present study, the field experiments in the two stations were approved by HAU. Further, the stations where field studies were conducted are not protected locations for endangered or protected species.

### Plant materials

The four-way cross mapping population including 305 individuals was developed from the four-way cross among D276/D72//A188/Jiao51. The four parental lines were selected based on the agronomic performances for a range of traits in maize breeding programs. All 305 individuals were self-crossed to develop progeny families. Twenty eight out of 305 individuals lacked enough self-pollinated seeds, and finally, 277 four-way cross F_1_ individuals were genotyped for genetic map construction, and their selfed progeny, known as four-way cross families, were used for phenotyping [6].

### Field trials and trait evaluation

In 2010, the 277 four-way cross families, together with their four parents were planted at the Jiyuan Experiment Station and Zhengzhou Experiment Station, respectively. Field experiments in each location were arranged in a randomized complete block design with three replicates. Each plot included one row with 4 m long and 0.67 m wide, and was overplanted and then thinned to 15 plants per row at a density of 52,500 plants per hectare.

To determine whether flowering time (FT) affects the trade-off between kernel size, FT was investigated and recorded as the number of days from planting when 50% of the plants in a row were shedding pollen. At physiological maturity, eight consecutive plants from the center of each plot were harvested by hand for trait measurements. The ear traits were evaluated, which included ear row number (ERN) and kernel number per row (KNR). After ears were dried down to a constant weight, the kernels at the middle of the ears in each plot were shelled and bulked. Four kernel traits were measured, including 100-kernel weight (HKW), kernel length (KL), kernel width (KW) and kernel thickness (KT). HKW was estimated from the average of three measurements of the weight of 100 randomly selected kernels; KL, KW and KT were estimated by the average of three replicated measurements of 50 kernels randomly selected from the bulked kernels using electronic digital calipers.

### Phenotypic data analysis

Analysis of variance for phenotype data was performed using the General Line Model (Proc GLM) procedure in SAS software [47], and Fisher Least Significant Different (LSD) method was used for multiple comparisons. The components of variance were estimated using a random-effect model and broad-sense heritability (*H*^2^) for each trait across the two environments was calculated as defined by Knapp et al. [48]. Phenotypic correlations among traits were calculated by the Pearson correlation method using the mean values of genotypes across environments.

### Genetic map and QTL mapping

Genetic linkage map was constructed using the algorithm proposed by Zhang et al. which was implemented in software package GACD as functionality CDM [49]. Two hundred and twenty one markers were relatively evenly distributed on 10 maize chromosomes and the whole length of the genome was 1799.03 cM [6].

The algorithm of inclusive composite interval mapping (ICIM) for four-way crosses was implemented in GACD software (http://www.isbreeding.net) as functionality CDQ [50] and used for QTL mapping of six traits, i.e., KL, KW, KT, HKW, KNR and ERN. QTL analysis was performed on the mean values of each genotype across the two environments. Inclusive linear models that includes marker variables and marker interactions so as to completely control both additive and dominance effects were built respectively for each trait. Stepwise regression was used to select significant marker variables and then used for background control in Inclusive Composite Interval Mapping (ICIM) of QTL [50]. The two probabilities for entering and removing variables were set at 0.001 and 0.002. The scanning step was 1 cM. LOD threshold was set at 3.97 by the empirical formula derived from Zhang et al. [50]. The original genotypes, phenotypes and linkage maps of the four-way cross population was available in S1 Dataset.

## Results

### Phenotypic variation and heritability

The phenotypic variations of kernel size and related traits among the four parental lines were investigated in Jiyuan and Zhengzhou locations in 2010, and significant variations were observed for all traits measured in this study, including three kernel size traits (i.e., KL, KW and KT), ear traits (i.e., ERN and KNE) and kernel weight (HKW) (Table 1). Among the four-way families comprising of 277 entries, extensive phenotypic variation was observed in kernel size, HKW, ERN, KNR as well as FT (Table 2, S1 Fig). The heritability (*H*^2^) of the traits ranged from 0.70 (KNR) to 0.81 (FT) across the two environments, suggesting that genetic factor played an important role in the four-way cross population.

**Table 1:** Summary of phenotype analysis and multiple comparisons of traits evaluated for the four parental lines.

Traits^a^	Lines	Mean	SD	Range	LSD 0.05	LSD 0.01
KL (mm)	Jiao51	7.68	0.56	6.07–9.44	a	A
	A188	8.70	0.61	7.05–9.78	b	B
	D276	9.62	0.71	8.13–11.34	c	C
	D72	9.87	0.66	8.26–11.08	d	C
KW (mm)	D276	6.93	0.64	5.25–8.96	a	A
	A188	7.48	0.52	5.97–8.74	b	B
	Jiao51	8.18	0.59	6.65–9.58	c	C
	D72	8.42	0.68	6.02–10.03	d	D
KT (mm)	A188	3.96	0.40	3.31–5.75	a	A
	Jiao51	5.00	0.51	3.71–6.26	b	B
	D276	5.31	0.55	3.93–6.94	c	C
	D72	5.81	0.75	6.20–7.39	d	D
ERN	A188	11.3	0.76	10.0–12.0	a	A
	D72	12.5	0.87	11.2–14.0	ab	AB
	D276	13.7	1.50	11.0–15.7	b	B
	Jiao51	16.1	0.62	15.2–17.0	c	C
KNR	D276	10.5	3.18	7.0–14.0	a	A
	D72	12.0	1.83	9.6–15.0	ab	A
	A188	12.5	1.93	10.0–14.5	b	A
	Jiao51	16.8	0.90	15.4–17.9	c	AB
HKW (g)	A188	9.3	1.01	7.58–10.5	a	A
	Jiao51	14.5	0.41	13.8–15.2	b	B
	D276	18.3	3.00	13.42–22.5	c	C
	D72	23.1	0.90	21.8–24.15	d	D

^a^: KL: kernel length; KW: kernel width; KT: kernel thickness; ERN: ear row number; KNR: kernel number per row; HKW: hundred kernel weight. LSD: Least Significant Difference; The same letters in LSD 0.05 and LSD 0.01 columns indicate that difference is not significant in the same group at P < 0.05 (LSD 0.05) or P < 0.01 (LSD 0.01) levels.

**Table 2:** Phenotypic variation among four-way cross families for all traits measured.

Traits	Min	Max	Mean±SD	*H*^2^
Flowering Time (days)	59.20	67.40	63.48±1.45	0.81
Kernel length (mm)	8.95	10.94	9.88±0.34	0.77
Kernel width (mm)	7.70	9.28	8.32±0.29	0.73
Kernel thickness (mm)	4.10	5.70	4.90±0.02	0.79
Ear row number	12.30	17.00	14.56±0.93	0.75
Kernel number per row	28.79	51.80	41.77±4.08	0.70
100 kernel weight (g)	19.55	29.95	23.46±1.85	0.71

Minimum (Min), maximum (Max) phenotypic values for each trait, as well as the phenotypic mean plus or minus their standard deviation (SD) and their broad-sense heritability (*H*^2^) were shown.

### Correlation of seed size and other traits

Of the traits surveyed in this study, a number of significant pairwise correlations were observed between kernel size and the other traits (i.e., FT, ERN, KNR and HKW) (Table 3). For example, ERN showed significant positive correlation with KL (*r* = 0.248), while with significant negative correlation with KW (*r* = -0.319) and KT (*r* = -0.187), suggesting the trade-offs between ERN and kernel size. However, KNR was only significantly correlated with one of the three traits of kernel size (KT, *r* = -0.606), which implied the trade-offs between KNR and KT instead of KL and KW. Significant positive correlations were also observed between HKW and kernel size (*r* values were 0.462, 0.693 and 0.493 for KL, KW and KT, respectively), which means each of the three kernel size components contributed to the weight of the kernel in this population. Correlation between FT and kernel size was observed, however, FT was significant but weak correlated with KL (*r* = 0.156), and not significant with KW and KT.

**Table 3:** Pairwise Pearson’s correlations between traits measured.

Traits^a^	FT	KL	KW	KT	ERN	KNR
KL	0.156*					
KW	-0.051	0.545**				
KT	-0.036	-0.176*	0.234**			
ERN	0.177**	0.248**	-0.319**	-0.187**		
KNR	-0.069	0.036	-0.095	-0.606**	0.043	
HKW	-0.107	0.462**	0.693**	0.493**	-0.196**	-0.260**

^a^: FT: flowering time; KL: kernel length; KW: kernel width; KT: kernel thickness; ERN: ear row number; KNR: kernel number per row; HKW: hundred kernel weight;

*: Significant at 0.05 level;

**: Significant at 0.01 level.

Given the extensive correlation among all traits, multiple linear regression model was used to estimate the effect of the different life-history traits on kernel size. The best fit model (smallest AIC) of KL (*F* = 54.53, *p*-value <2.2e^-16^, *r*^2^ = 0.56) included: KW, KT, ERN and HKW, which explained 15.9, 9.0, 8.1 and 16.3% of the variation of KL, respectively. For the KW, the best model (*F* = 103.4, *p*-value <2.2e^-16^, *r*^2^ = 0.65) could explain 65% of the variance. The model included: KL, ERN and HKW, which explained 19.2, 16.1 and 16.9% of the variation of kernel width, respectively. Similarly, KL, KNR and HKW can explain 13.2, 10.9 and 11.8% of the variation of KT, respectively (*F* = 47.0, *p*-value <2.2e^-16^, *r*^2^ = 0.45). Thus, agronomic traits can explain some of the variation in kernel size, but the variance explained is smaller than the heritability.

### QTL mapping results of kernel size and related traits

A summary of the QTL detected across environments, including the positions, LOD scores, genetic effects (additive effects of *a*_*F*_ and *a*_*M*_ and dominance effect *d*), phenotypic variation explained (PVE) and the mean values of four different genotypes, were shown in Table 4. A total of 10 QTL were identified for kernel size, including 5 QTL for KL, 3 QTL for KW and 2 QTL for KT. Single QTL of kernel size explained from 5.51% to 17.94% of the phenotypic variation. Five QTL were identified for HKW which located on chromosomes 1, 3, 5 and 7, and single QTL explained from 6.62% to 8.23% of the phenotypic variation. Six QTL were identified for KNR, which included 4 QTL on chromosome 5 and 1 each on chromosomes 1 and 3. Single QTL of KNR can explain from 5.13% to 6.85% of the phenotypic variation. Seven QTL were identified for ERN which located on chromosomes 1, 4, 6, 7, 9 and 10, and the largest QTL for ERN was located on chromosome 6 and explained 11.24% of the phenotypic variation.

**Table 4:** Estimated QTL locations and genetic effects affecting six traits using average data from two environments.

							Genetic effects^b^			
Traits^a^	QTL	Bin	Position (cM)	Left marker	Right marker	LOD	*a*_*F*_	*a*_*M*_	*d*	PVE (%)^c^
KL	*qKL3-1*	3.04/05	72	umc1347	bnlg1957	4.79	-0.03	-0.06	-0.04	7.41
	*qKL5-1*	5.06	129	umc1680	umc1019	12.13	0.01	0.14	0.01	17.94
	*qKL7-1*	7.02/03	78	bnlg1792	umc1567	4.87	0	-0.06	-0.07	7.41
	*qKL7-2*	7.03/04	121	umc1408	dupssr13	5.97	-0.09	-0.03	0.01	8.14
	*qKL10-1*	10.04/05	50	umc1053	umc1506	4.04	-0.07	-0.02	-0.02	5.51
KW	*qKW5-1*	5.03/04	61	bnlg1700	umc2298	4.84	0.08	0	0.03	9.15
	*qKW6-1*	6.00/01	0	phi126	umc1018	4.23	-0.01	0.06	-0.05	7.14
	*qKW7-1*	7.00	42	umc1642	bnlg2132	4.77	-0.07	0.04	0	9.54
KT	*qKT1-1*	1.07/08	131	bnlg1556	phi039	6.19	-6.24	-4.22	3.35	12.81
	*qKT5-1*	5.01	7	umc1766	umc1365	6.24	7.20	0.43	3.19	11.12
HKW	*qHKW1-1*	1.06/07	114	umc1590	bnlg1556	4.09	-0.47	-0.07	0.11	6.90
	*qHKW1-2*	1.11	233	phi227562	bnlg1006	4.18	-0.42	0.38	-0.10	6.72
	*qHKW3-1*	3.04	70	umc1012	umc1347	4.58	0.30	-0.08	-0.35	6.62
	*qHKW5-1*	5.03/04	61	bnlg1700	umc2298	6.15	0.46	0.13	0.18	8.23
	*qHKW7-1*	7.05	157	umc2222	phi082	4.77	-0.11	-0.36	-0.28	6.72
KNR	*qKNR1-1*	1.02	33	bnlg1429	bnlg1007	4.18	-0.55	0.93	0.03	6.22
	*qKNR3-1*	3.04	67	umc1012	umc1347	4.43	-0.19	-1.02	0.08	6.85
	*qKNR5-1*	5.01	0	umc1766	umc1365	5.03	-0.91	-0.53	-0.12	5.83
	*qKNR5-2*	5.03/04	54	bnlg1700	umc2298	4.55	-0.19	-0.94	-0.33	6.09
	*qKNR5-3*	5.04/05	70	umc1591	umc1348	4.27	-0.86	-0.31	-0.15	5.42
	*qKNR5-4*	5.07/08	179	bnlg2305	zct389	4.35	0.47	-0.80	0.07	5.13
ERN	*qERN1-1*	1.10/11	206	bnlg1347	umc2100	4.91	-0.18	-0.05	-0.05	5.10
	*qERN1-2*	1.11	235	phi227562	bnlg1006	4.06	0.19	-0.17	-0.04	4.87
	*qERN4-1*	4.08/09	121	umc2286	umc1051	4.11	-0.02	0.19	0.11	5.19
	*qERN6-1*	6.02/03	36	umc1656	umc1887	9.4	-0.04	-0.30	-0.01	11.24
	*qERN7-1*	7.02	76	phi034	bnlg1792	5.63	0.04	-0.24	0.04	5.94
	*qERN9-1*	9.03	79	umc1700	umc1691	4.59	-0.15	-0.13	0.02	4.74
	*qERN10-1*	10.01/02	13	umc1319	umc1576	5.61	0.22	0.01	-0.07	5.97

^a^: Trait abbreviation as follow, KL: kernel length; KW: kernel width; KT: kernel thickness; ERN: ear row number; KNR: kernel number per row; HKW: hundred kernel weight.

^b^: The genetic effects of *a*_*F*_ and *a*_*M*_ were the additive genetic effects of the two single crosses, D276×D72 and A188×Jiao51, respectively; the genetic effect of *d* was the dominance effect between the two single crosses.

^c^: Phenotypic variation explained.

**Fig 1:**
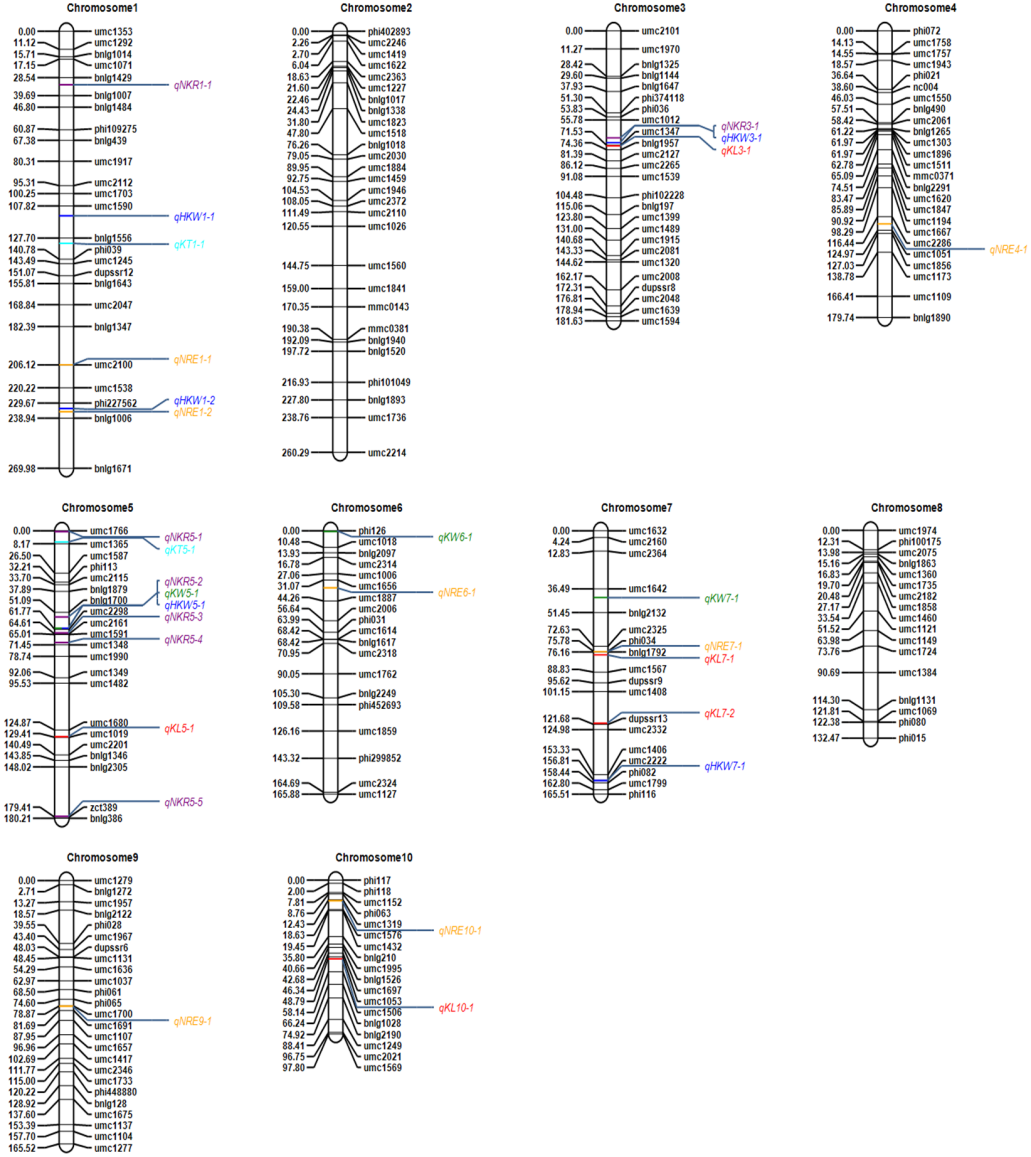
Genetic linkage maps and QTL identified in the four-way cross population.

Positions of all detected QTL were marked in the linkage maps, and overlaps between QTL of kernel size with other traits were observed (Table 4 and Fig 1). The first overlapped QTL located on chromosome 5 (bin 5.03/04). In this region, *qKW5-1*, which conferred the kernel width, shared the same flanking markers with *qHKW5-1* and also with *qKNR5-2*. The second region located on chromosome 3 (bin 3.04/05). In this region, *qHKW3-1* shared the same flanking markers with *qKNR3-1*, which had the largest effect for KNR. Moreover, one QTL for KL, *qKL3-1*, was also detected, which shared the same flanking marker umc1347 with *qHKW3-1* and *qKNR3-1*. Other region with closely linked QTL was also identified on chromosome 7 (bin 7.02/03). In this region, both QTL for KL (*qKL7-1*) and ERN (*qERN7-1*) were identified, and they shared the same marker bnlg1792 within the QTL region. Despite the significant correlations between kernel size traits (i.e., KL, KW and KW), we did not detect any overlapping QTL region for the three kernel-size traits.

## Discussion

### The trade-off between kernel size traits in maize

Grain seed is a life-history trait, and the trade-off of grain seed and related traits has widely reported in many plant species [2,37,44,45]. However, few studies have addressed the genetic mechanism behind trade-off of the factors involved in maize kernel development by taking into account life-history traits. In the present study, complex genetic mechanism behind the trade-off of seed traits in the four-way cross population was observed. On one hand, overlapped QTL for kernel size (i.e., *qKL3-1*, *qKL7-1* and *qKW5-1*) and yield-components were observed, and most of them had the same direction of additive effects (*a*_*F*_ and *a*_*M*_) (Table 4), which indicated the allele that increases kernel size is from the same natural accession, indicating past occurrence of directional selection for kernel size and yield components. On the other hand, kernel size (i.e., KW and KT) showed significant negative correlation with ERN and KNR (Table 3), which implied the potential trade-off behind them. However, there is little evidence for overlap in their genetic architecture since no common QTL between the traits were detected.

### Comparison with published QTL/gene

In the present study, we mapped 10 QTL for kernel size, with three of them (*qKW5-1*, *qKL3-1* and *qKL7-1*) had consistent co-localization or adjacent to QTL for one of the components of maize yield (Table 4). We compared the QTL with published kernel-size QTL, and overlapped QTL independent of the genetic background were identified.

*qKW5-1* with flanking markers bnlg1700 and umc2298 in the present study, shared the same QTL region with *CQTL5-1*, an common QTL for kernel width in multiple connected RIL populations in maize [8]; In this region, the other QTL for kernel width (i.e., *qKW5*) was also identified in an independent QTL mapping of kernel-size [9] (S1 Table). More importantly, we identified *qKW5-1* overlapped with the *qHKW5-1* (the QTL with the largest effect for 100-kernel weight in the present study), similar results were also observed by Li et al. [8] and Liu et al. [9], who also identified the QTL for kernel-width overlapped with kernel weight in this region. Within *qKW5-1* region, *ZmGW2-Chr5* conferring kernel size in maize has been identified and is perhaps one of candidate genes for the QTL [51]. Therefore, it could be concluded that this genomic region is very important for grain yield since the QTL has the stable expression across different genetic background.

*qKL7-1* located in bin 7.02/03 is another important region for the genetic control of grain yield and kernel traits. In this region, cluster QTL for kernel-size and yield-related trait were also identified in independent studies. For example, Li et al. [8] found one common QTL (*CQTL7-1*) conferring kernel weight, kernel width and thickness in multiple connected RIL populations in maize. Peng et al. [7] identified two QTL, *Qqknpp7* and *Qqgypp7*, conferring kernel number and grain yield per plant, respectively. Other kernel-size QTL in the present study with known QTL/gene included *qKL5-1* with *gln1-3* [20] and *Yqknpp5* [7], *qKT1-1* with *CQTL1-2* [8], and *qKT5-1* with *qKT5-1* [9]. The consistency of QTL/gene in independent study implied the common genetic basis for these traits (S1 Table).

### Joint analysis for multiple related traits

In this study, QTL analysis was performed on the six traits, respectively. In total, 10 QTL for kernel size (KL, KW and KT) and 18 QTL for other three traits (ERN, KNR and HKW) were detected. In fact, these traits were highly related (Table 3). Multiple-trait analysis took into account the correlated structure of multiple traits, and could improve the statistical power of the QTL detection and the precision of parameter estimation [52]. However, there were seldom studies focused on the QTL mapping methods for jointly analyzing multiple traits in four-way crosses till now on. We will try to develop a statistical method for joint analysis on four-way cross populations in the future.

### Implications for molecular-assisted selection (MAS) breeding

In the present study, a total of 10 significant QTL for kernel size were identified, which ranged from two for KT to five for KL. However, these QTL seemed to be independent genetic regulation of seed size since no consistent QTL were observed. These QTL could be valuable because it means that improvement in one trait can be accomplished without a corresponding decrease in the other. Here, we also found that at least three QTL (i.e., *qKL3-1*, *qKL7-1* and *qKW5-1*) with stable expression across kernel size and at least one of the other kernel related traits, and they had the same direction of the additive effects. These QTL may imply the genetic regulation of seed size and the components of maize yield, and may have high values using MAS to improve yield in maize.

## Supporting Information

S1 Fig The distributions of the seven traits across the two environments. A, kernel length; B, kernel width; C, kernel thickness; D, 100 kernel weight; E, number of rows per ear; F, number of kernels per row; G, flowing time.
(TIF)

S1 Dataset The original genotypes, phenotypes and linkage maps data of the four-way cross population used in this study. (CDQ)

S1 Table Comparison of the QTL identified in the present study with previous reported QTL/genes from the literature. (DOCX)
