# Supplementary figures and images for: The Genetic Basis of Natural Variation in Kernel Size and Related Traits Using a Four-Way Cross Population in Maize
Source: PLoS One. 2016 Apr 12;11(4):e0153428. doi: 10.1371/journal.pone.0153428 (PMC4829245; doi:10.1371/journal.pone.0153428)

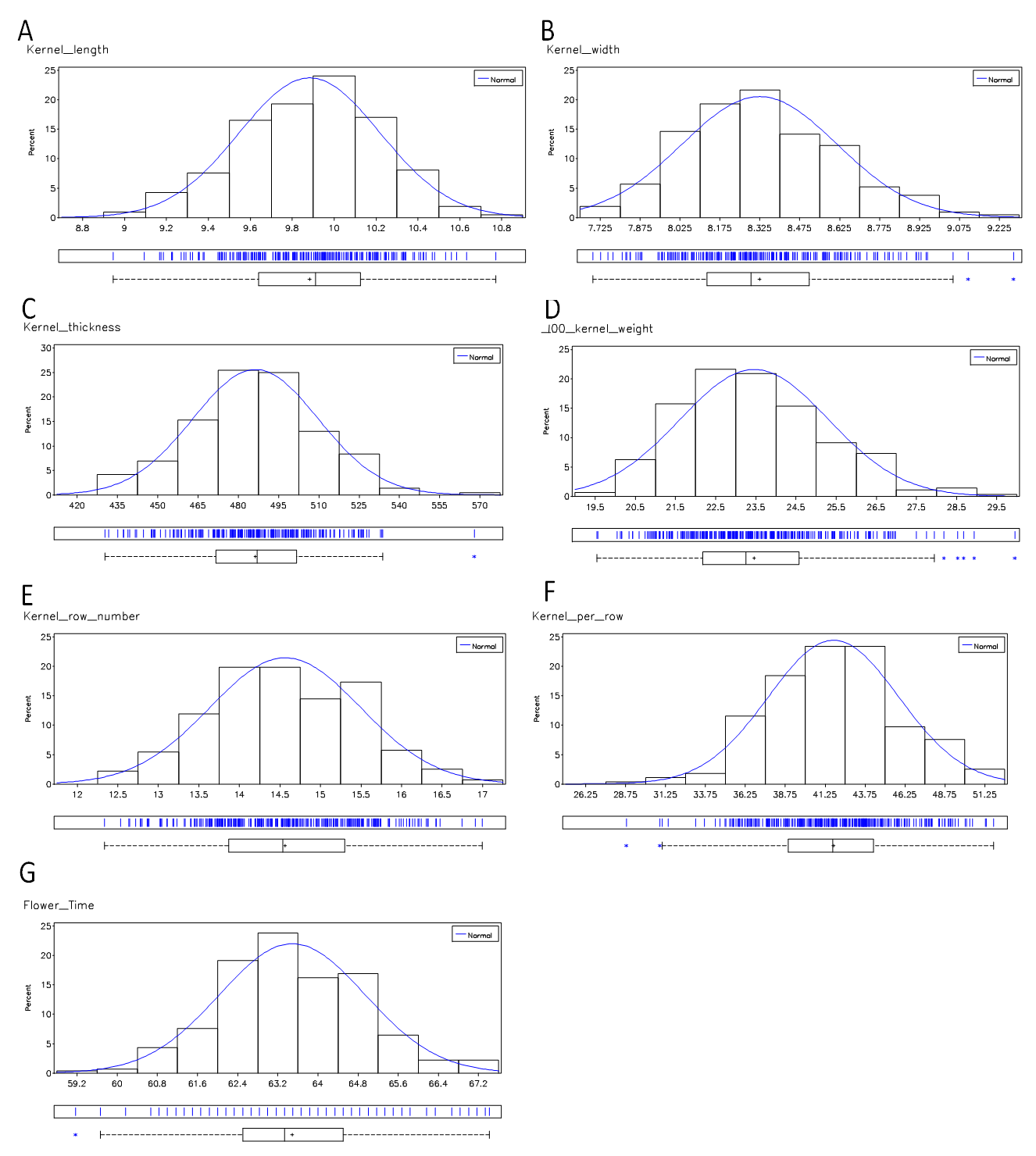

Supplement: S1 Fig — A, kernel length; B, kernel width; C, kernel thickness; D, 100 kernel weight; E, number of rows per ear; F, number of kernels per row; G, flowing time. (TIF) [file pone.0153428.s001.tif]
