# Supplementary material for: The Genetic Basis of Natural Variation in Kernel Size and Related Traits Using a Four-Way Cross Population in Maize
Source: PLoS One. 2016 Apr 12;11(4):e0153428. doi: 10.1371/journal.pone.0153428 (PMC4829245; doi:10.1371/journal.pone.0153428)
Supplement: S1 Table — (DOCX) [file pone.0153428.s003.docx]

**S1 Table.** Comparison of the QTL identified in the present study with previous reported QTL/genes from the literature.

| Traits^a^ | QTL | Bin | Left marker | Right marker | Reported genes/QTL |
| --- | --- | --- | --- | --- | --- |
| KL | *qKL3-1* | 3.04/05 | umc1347 | bnlg1957 | - |
|  | *qKL5-1* | 5.06 | umc1680 | umc1019 | *gln1-3*[20]*; Yqknpp5*[7] |
|  | *qKL7-1* | 7.02/03 | bnlg1792 | umc1567 | *CQTL7-1*[8]*; Qqgypp7*[7]*; Qqknpp7*[7] |
|  | *qKL7-2* | 7.03/04 | umc1408 | dupssr13 | *-* |
|  | *qKL10-1* | 10.04/05 | umc1053 | umc1506 | *-* |
| KW | *qKW5-1* | 5.03/04 | bnlg1700 | umc2298 | *zmGW2-Chr5* [51]*; CQTL5-1*[8]*; qKW5*[9]*; qHKW5*[9] |
|  | *qKW6-1* | 6.00/01 | phi126 | umc1018 | *-* |
|  | *qKW7-1* | 7.00 | umc1642 | bnlg2132 | *-* |
| KT | *qKT1-1* | 1.07/08 | bnlg1556 | phi039 | *CQTL1-2*[8] |
|  | *qKT5-1* | 5.01 | umc1766 | umc1365 | *qKT5-1*[9] |

^a^: KL: Kernel length; KW: Kernel width; KT: Kernel thickness.
